# The Baby as a Water-Drinker

**Published:** 1886-10-20

**Authors:** C. A. Bryce

**Affiliations:** Richmond, Va.


### THE BABY AS A WATER-DRINKER.

BY C. A. BRYCE, M.D., RICHMOND, VA.

In my earlier years of practice I devoted much attention to the study and treatment of infantile disorders, and have never regretted any labor or pains spent in the direction of ameliorating the ills of this large class of little sufferers. It would doubtless surprise many of my readers if I were to state that many infants annually die for the want of water—pure drinking water!—and still the assertion is true. Let me invite the attention of mothers and nurses to the fact that three-fourths of a child's nourishment for the first twelve months of its existence is, or should be, water. It is useless for me to explain just how oxygen is supplied to the tissues by this water, how the blood circulates by the presence of this water, how the impurities of the blood are carried along in a watery menstruum and eliminated by skin and kidneys through the medium of water. Physicians know this, and mothers hardly need to be told that such is a fact. It should be apparent, then, that this agent is a most important one in, as well as out of, a child's economy. Our paper shall not be a long one—but our practical application is—never allow a child to suffer for so important an agent as water. In fevers and inflammations it is cruel to withhold this health-giving beverage, upon the principle that the disease will be made worse by the use of water. There is no disease in which water should be withheld. I have seen a little infant with cholera-infantum, with dry husky skin, parched tongue, rolling and whining feebly, and almost in a state of collapse from frequent watery discharges, go into a sweet sleep, have a soft, velvety skin, a cessation of diarrhœa and a speedy recovery ensue from the free use of *spoonful doses of iced water*, when all other agents had failed. Do not imagine, mothers and nurses (and doctors also), that the watery discharges are due to too much water in the child's system. This is not the cause of your cholera infantum. But, unless you supply this waste of water, your little patient will die *from the want of it.* I have a lively recollection of a case such as I have described, which occurred quite a number of years ago in my practice, in which I called my esteemed friend, Dr. Jno. G. Skelton, of this city, in consultation. The doctor approved of all I had done in the case, and had nothing to suggest—but taking a saucer of cracked ice and a spoon he approached the infant (about nine months old) and offered it a spoonful of the iced water. As soon as it touched the lips of the little patient it swallowed it eagerly, and it never took its eyes off the doctor as long as he had the saucer and spoon in his hands. After drinking enough it fell into a sweet sleep and soon recovered. We need not mention the fact of the extreme fatality of genuine cholera in the adult, and would call the attention of all to the condition of these patients in the last stage of this disease. It is one of absolute loss of all the watery portion of the blood. My friend Dr. H. W. Davis, of this city, tells me of his treatment of a few cases of this disease on common-sense principles (ice water in it) by determining not to let them die from the want of water. He supplied them with iced water by the bucket-full, and the result was a cure of several hopeless cases! What is true of the adult is true of the child. Water, plentifully used, will never kill——will always afford comfort, and will many times save life.—*So. Clinic.*

